# Prognostic Value of p53 Status in Endometrial Cancer: Real-World Evidence from a Tertiary Center

**DOI:** 10.3390/cancers18111805

**Published:** 2026-06-01

**Authors:** Prapaporn Suprasert, Tanadon Salakphet, Tip Pongsuvareeyakul, Surapan Khunamornpong

**Affiliations:** 1 Division of Gynecologic Oncology, Department of Obstetrics and Gynecology, Faculty of Medicine, Chiang Mai University, Chiang Mai 50200, Thailand; tanadon.s@cmu.ac.th; 2 Division of Gynecologic Pathology, Department of Pathology, Faculty of Medicine, Chiang Mai University, Chiang Mai 50200, Thailand; tang_tip@hotmail.com (T.P.); skhunamo@yahoo.com (S.K.)

**Keywords:** endometrial cancer, p53, molecular classification, prognosis, survival, real-world data, clinicopathologic factors

## Abstract

**Background**: p53 immunohistochemistry is widely used as a surrogate marker for molecular classification in endometrial cancer and is generally associated with aggressive tumor behavior. However, its independent prognostic value in real-world clinical settings remains uncertain. **Methods**: This retrospective cohort study included patients with histologically confirmed endometrial cancer treated at a tertiary center between 2015 and 2023. Patients with available p53 immunohistochemical results were classified as p53 wild-type or p53-abnormal status. Clinicopathologic characteristics were compared between groups. Survival outcomes, including overall survival (OS) and progression-free survival (PFS), were analyzed using the Kaplan–Meier method and compared with the log-rank test. Cox proportional hazards models were used to identify independent prognostic factors. Sensitivity analyses were performed to assess the impact of treatment-related variables. **Results**: A total of 132 patients were included. p53-abnormal tumors were associated with older age, high-grade non-endometrioid histology, and more frequent use of adjuvant therapy. In Kaplan–Meier analysis, p53-abnormal status was associated with poorer OS (5-year OS: 54.8% vs. 77.1%, *p* = 0.029), with a similar trend observed for PFS. However, in multivariable analysis, p53 status was not independently associated with survival. Instead, advanced stage and residual disease remained significant prognostic factors. Sensitivity analyses incorporating treatment-related variables yielded consistent results, confirming the lack of independent prognostic impact of p53. **Conclusions**: Although p53-abnormal status was associated with adverse clinicopathologic features and worse unadjusted survival, it was not an independent prognostic factor after adjustment. These findings suggest that the prognostic impact of p53 is context-dependent and largely mediated by established clinical factors, particularly tumor burden. Integrated clinicopathologic and molecular assessment remains essential for accurate risk stratification in endometrial cancer.

## Simple Summary

Endometrial cancer can be classified using molecular markers, including p53 status. Tumors with abnormal p53 expression are generally considered aggressive and are often associated with poor outcomes. In this real-world cohort, p53-abnormal tumors were linked to adverse clinicopathologic features and worse survival in univariable analysis. However, after adjustment for established clinical factors, p53 status was not an independent predictor of survival. Instead, stage and residual disease remained the strongest prognostic factors. These findings suggest that the prognostic impact of p53 is context-dependent and underscore the importance of integrating molecular and clinicopathologic information for accurate risk stratification.

## 1. Introduction

Endometrial cancer (EC) is one of the most common gynecologic malignancies worldwide and represents an increasing global health burden. According to recent global cancer statistics, EC is the fifth most frequently diagnosed cancer among women, with an age-standardized incidence rate (ASR) of approximately 8.4 per 100,000 women worldwide [1]. The incidence varies substantially across geographic regions, with the highest rates reported in North America (ASR 22.3 per 100,000), whereas lower but steadily increasing rates have been observed in Southeast Asia. In Thailand, the incidence of EC is approximately 6.9 per 100,000 women, and the number of cases has shown a continuous upward trend over recent decades [1]. This increase is partly attributed to population aging, changes in reproductive patterns, obesity, and improved detection of early disease [2].

In the past decade, the management of EC has undergone a significant transformation following the integration of molecular-based classification into routine clinical practice. The TCGA molecular classification framework classifies EC into four biologically distinct groups based on genomic alterations [3]. This classification model was subsequently adapted for clinical use through surrogate markers that can be detected using immunohistochemistry (IHC) and targeted molecular testing. Contemporary international guidelines and the World Health Organization (WHO) now support a stepwise molecular diagnostic algorithm to classify EC into four molecular subgroups: (1) DNA polymerase epsilon *(POLE*) ultramutated tumors, (2) mismatch repair-deficient tumors (dMMR), (3) p53-abnormal tumors, and (4) tumors with no specific molecular profile (NSMP) [4,5,6]. Recent ESGO–ESTRO–ESP guidelines have further refined the prognostic classification of EC by integrating molecular features into risk stratification and adjuvant treatment recommendations [5]. In addition, NSMP tumors are increasingly recognized as a heterogeneous subgroup that may be further stratified according to estrogen receptor expression and histologic grade, including NSMP ER-positive/low-grade and NSMP ER-negative and/or high-grade tumors [5,7]. These molecular categories have important prognostic implications and increasingly guide risk stratification and adjuvant treatment decisions [8]. However, despite growing adoption of molecular classification, implementation in routine clinical practice remains challenging in many institutions because of resource limitations, testing costs, infrastructure requirements, and variable access to comprehensive molecular diagnostics, particularly in real-world and low-resource settings [9,10].

The growing integration of molecular classification has also influenced the contemporary staging of EC. The revised FIGO 2023 staging system incorporates molecular classification into stage assignment and prognostic grouping, resulting in potential stage migration or “stage shifting” in selected patients [11,12]. For example, *POLE*-mutated tumors may be downstaged because of their favorable prognosis [13,14], whereas p53-abnormal tumors may be assigned to higher-risk categories despite limited anatomic disease extent [15]. This evolving staging paradigm reflects the increasing recognition that tumor biology contributes substantially to prognosis beyond conventional clinicopathologic factors [11].

The recommended diagnostic algorithm typically includes testing for pathogenic mutations in the exonuclease domain of *POLE*, evaluation of mismatch repair (MMR) protein expression, and assessment of p53 status by IHC. Additional biomarkers, such as estrogen receptor and progesterone receptor expression, may also provide supplementary prognostic information [4,5,8]. However, in real-world clinical practice, comprehensive molecular testing is not always feasible. In particular, *POLE* sequencing requires specialized molecular techniques and may be costly, limiting its availability in many healthcare settings, including resource-limited regions. Consequently, molecular classification in real-world practice is often incomplete or limited to selected biomarkers. The absence of complete molecular profiling may lead to under-recognition of specific molecular subgroups and could influence risk stratification, adjuvant treatment selection, and prognostic assessment [16]. As a result, patients managed without full molecular classification may receive treatment strategies that differ from those recommended by contemporary molecular-integrated guidelines, potentially affecting survival outcomes.

Among the available surrogate markers, p53 IHC is widely accessible, relatively inexpensive, and routinely interpreted by gynecologic pathologists. Importantly, p53 IHC has been shown to correlate strongly with *TP53* mutation status, demonstrating a reported specificity of approximately 94% and sensitivity of around 91% [17]. Three abnormal staining patterns have been described: (1) diffuse strong nuclear overexpression, (2) complete absence of staining (null pattern), and (3) unequivocal cytoplasmic staining (cytoplasmic pattern) [8,17]. These patterns are collectively classified as “p53-abnormal,” whereas tumors demonstrating variable, heterogeneous staining are considered to exhibit a wild-type pattern [18].

The p53-abnormal molecular subtype accounts for approximately 10–15% of EC cases and is most commonly associated with serous carcinoma and high-grade endometrioid tumors [18]. This subtype is characterized by aggressive clinical behavior, increased likelihood of advanced-stage disease at diagnosis, higher recurrence risk, and poorer survival outcomes compared with other molecular subgroups [8,18]. As a result, patients with p53-abnormal tumors are frequently considered candidates for intensified adjuvant treatment strategies, including chemotherapy or combined chemoradiotherapy.

Despite the established prognostic importance of p53-abnormal status as a high-risk molecular subgroup in endometrial cancer, challenges remain regarding how p53 findings should be interpreted and applied in settings where comprehensive molecular profiling is not routinely available. In many institutions, particularly in resource-limited environments, p53 IHC may represent the only accessible molecular marker. Therefore, evaluating the clinicopathologic correlates and prognostic impact of p53 status in real-world cohorts may provide valuable information to support risk stratification and treatment planning in such settings.

Accordingly, this study aimed to investigate clinicopathologic factors associated with p53 status in patients with endometrial cancer and to determine whether p53 expression independently predicts survival outcomes in a real-world cohort from a tertiary center in Northern Thailand.

## 2. Materials and Methods

### 2.1. Study Design and Patient Population

This retrospective cohort study was approved by the Institutional Ethics Committee of the Faculty of Medicine, Chiang Mai University, Chiang Mai, Northern Thailand (study code No. OBG-2568-0482/Research ID:0482). Clinical and pathologic data were retrospectively collected from medical records of patients with histologically confirmed endometrial cancer who underwent primary surgical treatment at Chiang Mai University Hospital between January 2015 and December 2023. Due to the retrospective nature of the study, formal sample size estimation was not performed. All eligible patients with available p53 immunohistochemistry (IHC) results during the study period were consecutively included in the analysis. Patients were categorized into two groups according to p53 status: p53 wild-type and p53-abnormal. Baseline clinicopathologic characteristics, treatment modalities, and clinical outcomes were compared between the two groups.

Surgical treatment generally included total hysterectomy with bilateral salpingo-oophorectomy and collection of peritoneal cytology. The extent of nodal evaluation, including pelvic and/or para-aortic lymph node sampling or dissection, was individualized according to disease risk profile, intraoperative findings, patient fitness, and contemporary treatment recommendations during the study period. Decisions regarding postoperative adjuvant therapy were determined through multidisciplinary team discussion based on clinicopathologic risk factors.

After completion of treatment, patients were scheduled for follow-up every 3 months during the first year, every 4 months during the second year, every 6 months during years 3–5, and annually thereafter. Follow-up evaluations included medical history and physical examination. Imaging studies were performed when recurrence or disease progression was clinically suspected.

### 2.2. Clinicopathologic Variables

Collected clinical data comprised age at diagnosis, body mass index (BMI), clinical presentation, underlying medical conditions, and history of other malignancies. Surgical variables included the type of surgery, nodal evaluation, and presence of residual disease after surgery. Pathologic variables comprised histologic subtype, depth of myometrial invasion, lymphovascular space invasion (LVSI), cervical involvement, lymph node metastasis, peritoneal cytology, and FIGO stage. All cases were retrospectively reclassified according to the 2023 FIGO staging system for endometrial cancer prior to analysis. Treatment-related variables included neoadjuvant chemotherapy, adjuvant radiotherapy, adjuvant chemotherapy, and combined modality treatment. Study outcomes included recurrence and death during follow-up.

### 2.3. Immunohistochemistry Testing

**Figure 1:**
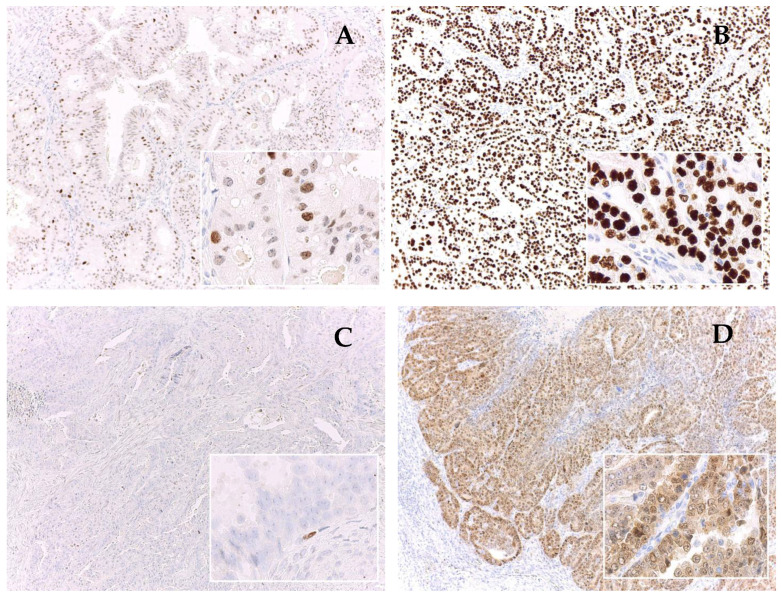
(**A**) Wild-type p53 expression showing heterogeneous nuclear staining with variable intensity in a subset of tumor cells. (**B**) Abnormal p53 expression (diffuse pattern) characterized by strong positivity in at least 80% of tumor nuclei. (**C**) Abnormal p53 expression (null pattern) characterized by the complete absence of tumor cell staining and preserved staining in internal control cells (inset). (**D**) Abnormal p53 expression (cytoplasmic pattern) characterized by unequivocal cytoplasmic staining of tumor cells accompanied by variable nuclear staining. Main images, original magnification ×100; inset images, original magnification ×400.

p53 status was determined by immunohistochemistry (DO-7 clone, DAKO, dilution 1:100) performed on tumor tissue specimens. Aberrant p53 expression patterns were classified as overexpression, null pattern, or cytoplasmic expression, whereas tumors showing heterogeneous staining were considered to exhibit a wild-type pattern. Representative examples of each p53 expression pattern are shown in Figure 1A–D. All p53 immunohistochemical results were independently reviewed by two gynecologic pathologists (S.K. and T.P.).

In routine clinical practice, complete molecular classification was not performed in all patients. Some patients underwent additional testing for MMR proteins or *POLE* mutation; however, these tests were not available for all cases. Therefore, molecular testing varied among patients, with some receiving p53 testing alone and others undergoing combined testing with MMR or *POLE* analysis.

### 2.4. Outcome Definitions

Progression-free survival (PFS) was calculated from the date of primary treatment initiation, defined as the date of surgery or the initiation of neoadjuvant chemotherapy when applicable, until the first documented evidence of disease recurrence or progression based on radiologic, pathologic, or clinical evaluation. Patients without progression events were censored at the date of last clinical follow-up. Overall survival (OS) was measured from the same starting point until death from any cause or last known follow-up. Follow-up information was collected through February 2026. Mortality data were cross-checked with the national population registry to ensure completeness of vital status ascertainment.

### 2.5. Statistical Analysis

Baseline clinicopathologic characteristics were summarized using descriptive statistical methods. Continuous variables were expressed as mean ± standard deviation (SD), and comparisons between groups were performed using the independent samples *t*-test. Categorical variables were presented as frequencies and percentages, with group comparisons conducted using either the chi-square test or Fisher’s exact test, as appropriate.

To identify clinicopathologic factors associated with p53-abnormal status, univariable logistic regression analysis was initially performed. Variables with *p* < 0.25 in univariable analysis and those considered clinically relevant were subsequently included in the multivariable logistic regression model using the enter method. Adjusted odds ratios (ORs) and 95% confidence intervals (CIs) were reported.

Survival outcomes, including progression-free survival (PFS) and overall survival (OS), were estimated using the Kaplan–Meier method and compared using the log-rank test. Univariable Cox proportional hazards regression analysis was initially performed to evaluate prognostic factors associated with OS. Variables with *p* < 0.25 in univariable analysis were subsequently entered into the multivariable Cox proportional hazards regression model to estimate adjusted hazard ratios (HRs) and 95% confidence intervals (CIs). Multivariable analyses were performed using complete-case data, and no imputation methods were applied because there were no substantial missing data among variables included in the final models. Statistical significance was defined as a two-sided *p* value < 0.05.

A sensitivity analysis was performed to evaluate the robustness of the survival model by additionally incorporating treatment-related variables into the multivariable Cox regression analysis. All statistical analyses were performed using IBM Statistical Package for the Social Sciences (SPSS) Statistics version 23.0 (IBM Corp., Armonk, NY, USA).

## 3. Results

### 3.1. Patient Characteristics and Correlates of p53 Status

A total of 132 patients were included, comprising 82 (62.1%) with p53-abnormal tumors and 50 (37.9%) with p53 wild-type tumors.

Patients with p53-abnormal tumors were significantly older than those with p53 wild-type tumors (mean age 64.9 ± 8.5 vs. 59.1 ± 9.1 years, *p* < 0.001). They more frequently presented with postmenopausal bleeding and had a higher prevalence of underlying comorbidities (*p* < 0.001). In addition, a history of other malignancies was more common in the p53-abnormal group (20.7% vs. 8.0%, *p* = 0.019).

Tumor characteristics also differed between groups. p53-abnormal tumors were strongly associated with non-endometrioid histology, particularly high-grade serous carcinoma (67.1% vs. 2.0%, *p* < 0.001), and were more likely to have positive peritoneal cytology (15.9% vs. 4.0%, *p* = 0.048). Patients in the p53-abnormal group were also more likely to receive adjuvant therapy (*p* < 0.001), reflecting a higher-risk clinical profile. However, no significant differences were observed in FIGO stage, depth of myometrial invasion, LVSI, or residual disease in univariable analyses (Table 1).

To further explore factors associated with p53-abnormal status, multivariable logistic regression analysis was performed. Older age (>60 years) (adjusted OR 7.83, 95% CI 3.17–19.36, *p* < 0.001), higher body mass index (≥25 kg/m^2^) (adjusted OR 3.22, 95% CI 1.30–7.96, *p* = 0.011), a history of other malignancies (adjusted OR 4.08, 95% CI 1.01–15.14, *p* = 0.036), and advanced stage (FIGO stage III–IV vs. I–II) (adjusted OR 2.78, 95% CI 1.02–7.54, *p* = 0.045) were independently associated with p53-abnormal tumors (Table S1), although FIGO stage, when analyzed as individual categories, was not significantly associated in univariable comparisons.

Molecular testing was not uniformly performed. Approximately 53.0% of patients underwent combined MMR and p53 testing (*n* = 70), while 40.9% underwent p53 testing alone (*n* = 54). Only a small proportion received more comprehensive molecular profiling, including MMR, p53, and *POLE* (3.8%, *n* = 5) or p53 and *POLE* (2.3%, *n* = 3). Among patients with additional molecular evaluation, heterogeneous molecular profiles were observed within the p53 wild-type group, with a substantial proportion classified as dMMR and only a minority harboring *POLE* mutations (Table S2).

### 3.2. Survival Outcomes According to p53 Status

During follow-up, recurrence occurred in 19 patients (23.2%) in the p53-abnormal group and 7 patients (14.0%) in the p53 wild-type group. Death was observed in 36 patients (43.9%) and 15 patients (30.0%), respectively.

Kaplan–Meier analysis demonstrated differences in survival outcomes according to p53 status. PFS tended to be lower in patients with p53-abnormal tumors compared with those with p53 wild-type tumors, with 5-year PFS rates of 65.3% versus 86.4%, although this difference did not reach statistical significance (log-rank *p* = 0.128) (Figure S1A).

Consistently, OS was significantly worse in the p53-abnormal group, with 5-year OS rates of 54.8% compared with 77.1% in the p53 wild-type group (log-rank *p* = 0.029) (Figure S1B).

**Figure 2:**
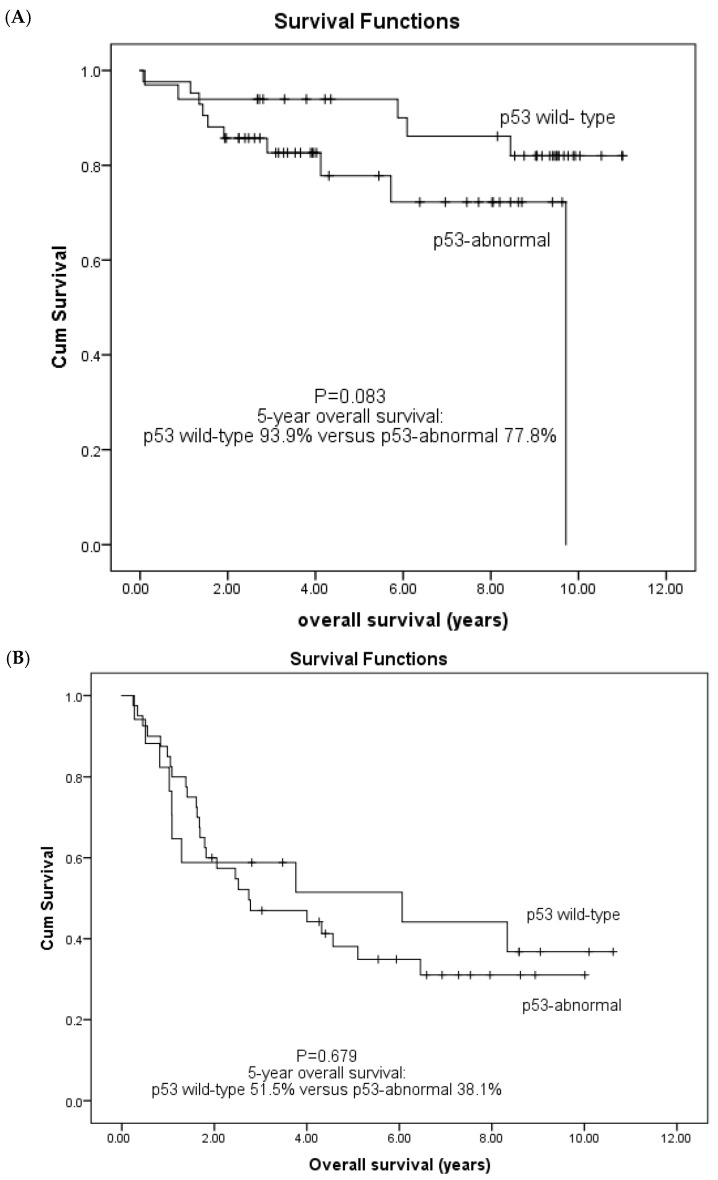
(**A**) Kaplan–Meier curves for overall survival in early-stage endometrial cancer according to p53 status. (**B**) Kaplan–Meier curves for overall survival in advanced-stage endometrial cancer according to p53 status.

When stratified by disease stage, a trend toward worse survival among patients with p53-abnormal tumors was observed in early-stage disease (5-year OS 77.8% vs. 93.9%, *p* = 0.083), whereas no significant difference was found in advanced-stage disease (5-year OS 38.1% vs. 51.5%, *p* = 0.679) (Figure 2A,B). Notably, in the early-stage subgroup, patients with p53-abnormal tumors were significantly more likely to receive adjuvant chemotherapy or combined chemoradiotherapy compared with those with p53 wild-type tumors (90.3% vs. 9.7%, *p* < 0.001).

In an exploratory analysis restricted to early-stage p53-abnormal tumors, no statistically significant difference in overall survival was observed according to receipt of adjuvant chemotherapy with or without radiotherapy, although a numerically higher 5-year overall survival was noted among those who received adjuvant treatment (76.2% vs. 66.8%, *p* = 0.631) (Figure S2).

Further exploratory analysis according to p53-abnormal subtypes (overexpression, null, and cytoplasmic patterns) did not demonstrate significant differences in overall survival (log-rank *p* = 0.545) (Figure S3).

### 3.3. Univariable and Multivariable Cox Regression Analyses (Table 2)

In univariable Cox regression analysis, several factors were associated with worse overall survival, including underlying disease (HR 2.27, 95% CI 1.10–4.70, *p* = 0.027), residual disease (HR 4.60, 95% CI 2.27–9.35, *p* < 0.001), deep myometrial invasion (≥50%) (HR 3.23, 95% CI 1.69–6.18, *p* < 0.001), high-grade tumor (HR 3.12, 95% CI 1.32–7.36, *p* = 0.009) positive peritoneal cytology (HR 4.02, 95% CI 2.03–7.96, *p* < 0.001), advanced stage (HR 4.30, 95% CI 2.35–7.89, *p* < 0.001), and p53-abnormal status (HR 1.97, 95% CI 1.060–3.65, *p* = 0.032).

However, after adjustment for clinically relevant factors in multivariable analysis, only advanced stage (adjusted HR 2.23, 95% CI 1.08–4.59; *p* = 0.029) and residual disease (adjusted HR 2.45, 95% CI 1.13–5.29; *p* = 0.020) remained independently associated with worse overall survival. p53 status was not independently associated with survival after adjustment (adjusted HR 0.78, 95% CI 0.34–1.81; *p* = 0.57).

**Table 2:** Univariable and multivariable Cox proportional hazards analyses of prognostic factors for overall survival in endometrial cancer (N = 132).

	Total (%)	5-Year OS (%)	Unadjusted HR (95% CI)	*p* Value	Adjusted HR * (95% CI)	*p* Value
Age (years)						
Less than or equal to 60	53 (40.2)	71.1	1.510 (0.841–2.709)	0.167	1.349 (0.704–2.586)	0.367
More than 60	79 (59.8)	60.0				
Body mass index (kg/m^2^)						
Less than 25	72 (54.5)	65.2	0.908 (0.523–1.578)	0.733	-	-
More than or equal to 25	60 (45.5)	65.2				
History of other malignancies						
None	111 (84.1)	65.4	1.045 (0.468–2.333)	0.914	**-**	**-**
present	21 (15.9)	66.7				
Underlying disease						
None	37 (28.0)	85.5	2.272 (1.098–4.703)	0.027	1.926 (0.883–4.201)	0.100
Present	95 (72.0)	58.9				
Residual disease						
None	119 (90.2)	70.2	4.602 (2.265–9.347)	<0.001	2.450 (1.135–5.286)	0.022
Present	13 (9.8)	30.8				
Myometrial invasion						
Less than 50%	58 (43.9)	85.9	3.227 (1.685–6.180)	<0.001	1.846 (0.909–3.749)	0.090
More than or equal to 50%	74 (56.1)	51.2				
Lympho-vascular space invasion						
None	76 (57.6)	71.9	1.310 (0.756–2.269)	0.335	-	-
Yes	56 (42.2)	59.0				
Tumor grade						
Grade 1&2	30 (22.7)	84.8	3.119 (1.322–7.357)	0.009	2.033 (0.690–5.988)	0.198
Grade 3	102 (77.3)	56.1				
Peritoneal washing						
Negative	117 (88.6)	70.6	4.018 (2.028–7.959)	<0.001	1.854 (0.886–3.879)	0.101
Positive	15 (11.4)	31.1				
p53						
Wild	50 (37.9)	79.7	1.966 (1.060–3.647)	0.032	0.784 (0.340–1.806)	0.567
Abnormal	82 (62.1)	56.9				
Figo Stage (2023)						
Early (I&II)	75 (56.8)	85.7	4.301 (2.348–7.877)	<0.001	2.232 (1.085–4.592)	0.029
Advanced (III&IV)	57 (43.2)	42.2				

OS, overall survival; HR, hazard ratio; 95% CI, 95% confidence interval. * Variables with *p* < 0.25 in univariable analysis were included in the multivariable Cox regression model using the enter method.

### 3.4. Sensitivity Analysis (Table S3)

In sensitivity analyses incorporating treatment-related variables, including neoadjuvant chemotherapy and adjuvant therapies, the overall findings remained nearly consistent. Advanced stage (adjusted HR 2.36, 95% CI 1.08–5.16, *p* = 0.031) and residual disease (adjusted HR 2.55, 95% CI 1.71–5.57, *p* = 0.018) remained associated with worse overall survival, whereas p53 status remained non-significant (adjusted HR 0.72, 95% CI 0.29–1.75, *p* = 0.466).

In this extended model, deep myometrial invasion was also significantly associated with survival. Adjuvant chemotherapy demonstrated a protective effect after adjustment (adjusted HR 0.27, 95% CI 0.10–0.71, *p* = 0.008), despite being associated with worse outcomes in univariable analysis (HR 1.98, 95% CI 1.10–3.59, *p* = 0.023). In contrast, adjuvant radiation with or without chemotherapy remained associated with worse survival in both univariable and multivariable analyses.

## 4. Discussion

In this study, p53-abnormal status was associated with poorer overall survival in univariable analysis; however, the association was attenuated after adjustment for clinicopathologic factors. In contrast, advanced stage and residual disease remained independently associated with worse survival, highlighting their strong prognostic influence in this cohort. Given the relatively small sample size, limited number of outcome events, and potential selection bias, the absence of an independent association should be interpreted cautiously and does not negate the established prognostic relevance of p53-abnormal status reported in larger molecular classification studies.

In our cohort, p53-abnormal tumors were more frequently observed in older patients and were strongly associated with high-grade, non-endometrioid histology, particularly high-grade serous carcinoma, consistent with an aggressive tumor phenotype. These findings are consistent with previous studies demonstrating that p53-abnormal status is enriched in biologically high-risk endometrial cancers, particularly high-grade serous tumors [3,19,20,21,22]. However, despite this association with adverse clinicopathologic features, p53 status did not retain independent prognostic significance after adjustment in our cohort. This result should be interpreted cautiously, as the relatively small sample size and limited number of outcome events may have reduced the statistical power to detect a modest independent prognostic effect of p53-abnormal status after multivariable adjustment.

In contrast, prior molecular classification studies have consistently demonstrated poorer outcomes among p53-abnormal tumors. The Cancer Genome Atlas (TCGA) demonstrated that copy-number high tumors, which largely correspond to p53-abnormal tumors, were associated with the poorest outcomes [3]. Subsequent studies validating TCGA-derived surrogate molecular classifiers, including the Proactive Molecular Risk Classifier for Endometrial Cancer (ProMisE) and TransPORTEC algorithms, also confirmed that p53-abnormal endometrial cancer represents an adverse prognostic subgroup [19,20,21]. The ProMisE classifier applies a hierarchical molecular classification approach using mismatch repair (MMR) status, *POLE* mutation analysis, and p53 immunohistochemistry as surrogate markers for TCGA molecular subgroups. In the PORTEC-3 molecular analysis, patients with p53-abnormal tumors had the poorest prognosis, with 5-year recurrence-free and overall survival rates of 48.0% and 54.0%, respectively [21]. Similarly, Bosse et al. [23] reported that molecular classification refined prognostic stratification among grade 3 endometrioid carcinomas, with p53-abnormal tumors showing unfavorable outcomes compared with other molecular subgroups. The final validation of the ProMisE classifier further supported its reproducibility and prognostic relevance in clinical practice [24]. More recently, a systematic review and meta-analysis including over 5000 patients confirmed that *TP53* alterations, defined by abnormal p53 expression and/or *TP53* mutations, were associated with significantly worse overall and disease-free survival [25].

However, not all studies have demonstrated an independent prognostic impact of p53 status after adjustment for clinicopathologic variables. A recent retrospective study by Elçiçek et al. [26] evaluating prognostic factors for recurrence in endometrial cancer found that p53-abnormal status was associated with recurrence in univariable analysis but did not remain significant in multivariable analysis, whereas established clinicopathologic risk stratification retained independent prognostic significance. Our findings are consistent with this observation and suggest that the prognostic effect of p53-abnormal status may be partly mediated through its association with other adverse clinicopathologic features, particularly advanced stage, high-grade histology, and residual disease.

Several factors may explain these differences. First, the strong prognostic influence of established clinicopathologic factors, particularly stage and residual disease, may attenuate the apparent contribution of molecular classification in real-world settings, as reflected by the consistent effect of stage across all models in this cohort [5]. In addition, the relatively small sample size, limited number of outcome events, and adjustment for correlated adverse prognostic variables may have reduced the statistical power to detect an independent effect of p53 abnormal status after multivariable adjustment. The observed association of stage in multivariable analysis, despite non-significance in univariable comparisons, may also reflect the effect of dichotomization (early vs. advanced stage) and adjustment for confounding variables.

Second, the p53 wild-type group likely represented a biologically heterogeneous population because comprehensive molecular classification was not uniformly available for all tumors. Although *POLE*-mutated tumors are relatively uncommon in non-endometrioid high-risk histology, incomplete molecular testing may still have resulted in limited misclassification within the p53 wild-type subgroup, particularly among endometrioid carcinomas. This is clinically relevant because *POLE*-mutated tumors are associated with excellent prognosis, whereas dMMR tumors generally demonstrate intermediate outcomes [20,22]. Consequently, residual molecular heterogeneity within the p53 wild-type group may have attenuated the observed prognostic contrast between p53 categories.

Another important consideration is the potential for referral and selection bias. During the study period, p53 immunohistochemistry was not routinely performed for all patients with endometrial cancer and was implemented gradually in routine clinical practice. Testing decisions were influenced by physician judgment, tumor characteristics, perceived risk profile, tissue availability, and resource considerations. As a result, patients with high-risk clinicopathologic features may have been more likely to undergo p53 testing, which could have affected cohort representativeness and interpretation of prognostic analyses.

In addition to tumor biology, treatment-related factors may also influence the observed survival patterns. In our cohort, patients with p53-abnormal tumors were substantially more likely to receive adjuvant chemotherapy compared with those with p53 wild-type tumors, reflecting their higher-risk clinical profile. In sensitivity analyses, adjuvant chemotherapy was associated with worse survival in univariable analysis but demonstrated a protective effect after adjustment. This pattern likely reflects confounding by indication, whereby patients receiving adjuvant chemotherapy had more aggressive disease at baseline, and the beneficial effect of therapy became apparent only after accounting for disease severity. In addition, differences in treatment adherence and completion of planned therapy may also have influenced survival outcomes in this retrospective real-world cohort.

In contrast, adjuvant radiation with or without chemotherapy remained associated with poorer survival even after multivariable adjustment. This finding should be interpreted with caution, as it likely reflects residual confounding, whereby patients selected for more intensive treatment harbored particularly high-risk disease characteristics that were not fully captured in the available clinicopathologic variables. Therefore, these results should not be interpreted as evidence of harm from adjuvant treatment, but rather as a reflection of underlying disease severity in a real-world setting.

This interpretation is partly supported by molecularly stratified analyses from the PORTEC trials. Horeweg et al. [27] demonstrated that patients with p53-abnormal tumors derived substantial benefit from external beam radiotherapy in the PORTEC-1 and PORTEC-2 trials, while updated results from the PORTEC-3 trial showed improved outcomes with adjuvant chemoradiotherapy compared with radiotherapy alone [28].

This pattern was also observed in the stage-stratified analysis. Although a trend toward worse survival was noted among patients with p53-abnormal tumors in early-stage disease, the difference did not reach statistical significance. This may have been related to the limited sample size and reduced statistical power within the stratified subgroup analysis. Notably, patients with p53-abnormal tumors in the early-stage subgroup were substantially more likely to receive adjuvant chemotherapy or combined chemoradiotherapy compared with those with p53 wild-type tumors. In an exploratory analysis restricted to early-stage p53-abnormal tumors, a numerically higher survival rate was observed among those who received adjuvant chemotherapy with or without radiotherapy compared with those who did not, although the difference was not statistically significant. This finding may reflect limited statistical power due to small sample size and low event rates and suggests that adjuvant treatment may partially mitigate the adverse prognosis associated with p53-abnormal tumors in early-stage disease.

Importantly, our findings should not be interpreted as evidence against the biological or clinical relevance of p53-abnormal status. Rather, the lack of independent significance in our cohort may reflect limited statistical power, incomplete molecular classification, and the strong association between p53-abnormal status and other adverse clinicopathologic features included in the multivariable models.

From a clinical perspective, these findings should be interpreted within the context of the study design and real-world setting. Although p53 immunohistochemistry is an established surrogate marker for molecular classification and an important component of risk stratification, its isolated prognostic impact may be more difficult to demonstrate in smaller retrospective cohorts with incomplete molecular profiling. Established clinicopathologic factors, particularly stage and residual disease, remained major determinants of outcome in our study. These findings support integrated models that combine molecular and clinicopathologic parameters rather than relying on a single biomarker alone, consistent with recent studies emphasizing the complementary prognostic value of molecular and conventional clinicopathologic assessment [25,29,30].

This study benefits from real-world data and comprehensive survival analysis, including multivariable and sensitivity models. However, several limitations should be acknowledged. Molecular testing was incomplete, precluding full molecular classification according to TCGA subgroups [3]. The retrospective design introduces potential selection and referral bias, as p53 immunohistochemistry was not routinely performed for all patients during the study period and was implemented gradually in real-world clinical practice. Testing decisions were influenced by physician discretion, tumor characteristics, tissue availability, and resource considerations. Consequently, the study cohort may not fully represent the overall endometrial cancer population treated at our institution. In addition, the number of patients undergoing comprehensive molecular testing, particularly for *POLE* mutations, was limited.

Approximately 30% of patients also did not undergo pelvic lymph node assessment. As nodal evaluation was performed selectively according to tumor risk factors, intraoperative findings, patient comorbidities, and prevailing guidelines at the time of treatment, some patients may have been understaged, particularly in the absence of sentinel lymph node mapping and ultrastaging. This may have influenced stage allocation and prognostic analyses. Furthermore, the relatively small number of outcome events may have reduced the statistical power to detect a modest independent effect of p53 status after multivariable adjustment. Finally, progression-free survival outcomes should be interpreted cautiously because progression events were retrospectively collected from routine clinical records and may have been influenced by variable follow-up patterns and informative censoring.

Exploratory analysis of p53-abnormal subtypes (overexpression, null, and cytoplasmic patterns) did not demonstrate significant differences in survival outcomes. While a previous study suggested that distinct p53 staining patterns may reflect different biological behaviors, our findings did not support this hypothesis [31]. This may be attributable to limited statistical power, particularly for rare subgroups, and therefore, these results should be interpreted with caution and warrant further investigation.

Future studies are warranted to further clarify the prognostic role of p53 within the context of integrated molecular classification. Prospective studies incorporating comprehensive molecular profiling, including MMR and *POLE* status, are needed to better define the independent contribution of p53 across molecular subgroups. In addition, studies evaluating treatment–biomarker interactions may help determine whether the prognostic impact of p53 is modified by adjuvant therapy. Such approaches may provide a more precise framework for risk stratification and personalized treatment in endometrial cancer.

## 5. Conclusions

In conclusion, p53-abnormal status was associated with poorer survival in unadjusted analyses, although this association was attenuated after adjustment for clinicopathologic variables in this cohort. Advanced stage and residual disease remained the dominant determinants of survival, underscoring the central role of tumor burden in patient outcomes. These findings support the integration of molecular and clinicopathologic factors for risk stratification in endometrial cancer rather than reliance on a single biomarker alone. However, given the retrospective design, incomplete molecular testing, potential selection and referral bias, and limited statistical power for multivariable analyses, the results should be interpreted with caution and should not be considered contradictory to the established prognostic significance of p53-abnormal status reported in larger molecular classification studies. Further studies incorporating comprehensive molecular profiling, including complete molecular classification, are warranted to better clarify the independent prognostic impact of p53 status in real-world settings.

## Figures and Tables

**Table 1:** Clinicopathologic characteristics of patients with endometrial cancer stratified by p53 status (N = 132).

	P53 Wild-Type (%)	P53-Abnormal (%)	*p* Value **
Total	50 (37.9)	82 (62.1)	
Mean age ± SD (years)	59.1 ± 9.1	64.9 ± 8.5	<0.001
Mean body mass index, kg/m^2^ (±SD):	24.6 ± 5.2	25.7 ± 4.8	0.221
Clinical presentation			0.049
Abnormal uterine bleeding	10 (20.0)	6 (7.3)	
Postmenopausal uterine bleeding	40 (80.0)	74 (90.2)	
Pelvic mass	-	2 (2.4)	
Underlying disease			<0.001
None	25 (50.0)	12 (14.6)	
DM & HT & DLP	2 (4.0)	14 (17.1)	
HT&DLP	1 (0.8)	10 (12.2)	
HT	6 (12.0)	8 (9.8)	
DM&HT	3 (6.0)	6 (7.3)	
Other *	13 (26.0)	32 (39.0)	
History of other malignancies			0.019
None	46 (92.0)	65 (79.3)	
Breast cancer	-	9 (11.0)	
Colon cancer	2 (4.0)	2 (2.4)	
Rectal cancer	-	2 (2.4)	
Cervical cancer	-	2 (2.4)	
Hepatocellular carcinoma	-	1 (1.2)	
Colon and breast cancer	-	1 (1.2)	
Ovarian cancer	2 (4.0)	-	
Outcome			
Recurrence	7 (14.0)	19 (23.2)	0.261
Death	15 (30.0)	36 (43.9)	0.141
Neoadjuvant chemotherapy	5 (3.8)	8 (9.8)	1.000
Nodal evaluation			
Paraaortic node sampling	22 (44.0)	19 (23.2)	0.019
Pelvic node dissection	39 (78.0)	52 (63.4)	0.085
Type of surgery			0.210
Laparotomy	43 (86.0)	77 (93.9)	
Laparoscopy	7 (14.0)	5 (6.1)	
Residual disease	5 (10.0)	8 (9.8)	1.000
Histology			<0.001
Endometrioid carcinoma	46 (92.0)	10 (12.2)	
High-grade serous cancer	1 (2.0)	55 (67.1)	
Carcinosarcoma	3 (6.0)	6 (7.3)	
Clear cell carcinoma	-	11 (13.4)	
Myometrial invasion			0.528
Endometrium	2 (4.0)	4 (4.9)	
Less than 50%	22 (44.0)	30 (36.6)	
More than or equal to 50%	17 (34.0)	24 (29.3)	
Subserosa	9 (18.0)	24 (29.3)	
Lymphovascular space invasion			0.292
None	19 (38.0)	34 (41.5)	
Focal	6 (12.0)	17 (20.7)	
Substantial	25 (50.0)	31 (37.8)	
Endocervical metastasis	11 (22.0)	26 (31.7)	0.240
Pelvic node metastasis	8/39 (20.5) ***	15/52 (28.8) ***	0.816
Paraaortic metastasis	3/22 (13.6) ***	3/19 (15.8) ***	0.673
Peritoneal washing positive	2 (4.0)	13 (15.9)	0.048
P53 type			
Overexpression	-	65 (79.3)	
Null	-	15 (18.3)	
Cytoplasmic	-	2 (2.4)	
FIGO stage (2023)			0.118
IA1	1 (2.0)	-	
IA2	7 (14.0)	-	
IB	5 (10.0)	-	
IC	-	3 (3.7)	
IIB	12 (24.0)	-	
IIC	8 (16.0)	39 (47.6)	
IIIA1	-	2 (2.4)	
IIIB1	-	3 (3.7)	
IIIC1i	4 (8.0)	10 (12.2)	
IIIC2i	2 (4.0)	2 (2.4)	
IVC	4 (8.0)	13 (15.9)	
Adjuvant treatment			<0.001
None	13 (26.0)	11 (13.4)	
Adjuvant radiation	20 (40.0)	9 (11.0)	
Adjuvant radiation and chemotherapy	11 (22.0)	33 (40.2)	
Chemotherapy	6 (12.0)	29 (35.4)	

* other: P53 wild-type: dyslipidemia (DLP) (1), hypothyroid (1), heart disease (1), end stage renal disease (ESRD) (1), hyperthyroidism & CA ovary (1), hypertension (HT) & DLP & coronary artery disease (CAD) (1), HT & DLP & chronic kidney disease (CKD) & CAD (1), HT & DLP & old cerebrovascular accident (CVA) (1), HT & CA colon (2), DM & asthma (1), DM & DLP (1), HT + valvular heart disease (VHD) (1). P53-abnormal: DM (1), DLP (1), heart disease (2), CA breast (5), ESRD (1), asthma (1), CA colon (2), hepatocellular carcinoma (1), CA cervix (2), HT & DLP & CAD (2), HT & VHD (2), DM & HT & ESRD (2), HT & gout (1), DM & HT & DLP & abdominal aortic aneurysm (1), DM&HT&CA breast&CA colon (1), DM & HT & DLP & oldCVA & CArectum (1), HT & CAbreast (4), DM&HT & triple vessel disease&old CVA (1), HT & DLP & CA rectum (1). ** *t*-test or chi-square or Fisher’s exact as appropriate.*** Percentages were calculated among patients who underwent nodal dissection. DM, diabetes mellitus; HT, hypertension; DLP, dyslipidemia.

## Data Availability

The data presented in this study are available on request from the corresponding author.

## Supplementary Materials

The following supporting information can be downloaded at https://www.mdpi.com/article/10.3390/cancers18111805/s1, Figure S1: Kaplan–Meier curves for survival outcomes according to p53 status. (A) Kaplan–Meier analysis of progression-free survival (PFS) according to p53 status. Patients with p53 wild-type tumors showed a trend toward longer PFS compared with those with p53-abnormal tumors; however, the difference did not reach statistical significance (log-rank test, *p* = 0.128). The estimated 5-year PFS rates were 86.4% for the p53 wild-type group and 65.3% for the p53-abnormal group. (B) Kaplan–Meier analysis of overall survival (OS) according to p53 status. Patients with p53 wild-type tumors demonstrated significantly improved survival compared with those with p53-abnormal tumors (log-rank test, *p* = 0.029). The estimated 5-year OS rates were 77.1% for the p53 wild-type group and 54.8% for the p53-abnormal group. Tick marks indicate censored observations; Figure S2: Overall survival according to adjuvant treatment in patients with early-stage p53-abnormal endometrial cancer. Kaplan–Meier curves showing overall survival among patients with early-stage p53-abnormal endometrial cancer according to receipt of adjuvant chemotherapy with or without radiotherapy. Patients receiving adjuvant treatment demonstrated a numerically higher 5-year overall survival compared with those who did not receive chemotherapy (76.2% vs. 66.8%); however, the difference was not statistically significant (log-rank *p* = 0.631); Figure S3: Kaplan–Meier curves for overall survival according to p53-abnormal staining patterns. Kaplan–Meier analysis of overall survival (OS) according to p53-abnormal immunohistochemical staining patterns, including overexpression, null, and cytoplasmic subtypes. No statistically significant differences in OS were observed among the three groups (log-rank test, *p* = 0.545). The estimated 5-year OS rates were 77.1% for the overexpression pattern and 56.6% for the null pattern, while the cytoplasmic subgroup demonstrated a 2-year OS of 100%. Tick marks indicate censored observations; Table S1:Clinicopathologic correlates of p53-abnormal status: univariable and multivariable logistic regression analyses; Table S2: Distribution of molecular testing and results in endometrial cancer (N = 132); Table S3: Sensitivity analysis: univariable and multivariable Cox proportional hazards analyses of prognostic factors for overall survival (N = 132).

## Abbreviations

The following abbreviations are used in this manuscript:

OS	overall survival
PFS	progression-free survival
EC	endometrial cancer
ASR	age-standardized incidence rate
TCGA	The Cancer Genome Atlas
IHC	immunohistochemistry
WHO	The World Health Organization
*POLE*	polymerase epsilon
dMMR	mismatch repair-deficient
NSMP	tumors with no specific molecular profile
MMR	mismatch repair
BMI	body mass index
LVSI	lympho-vascular space invasion
FIGO	International Federation of Gynecology and Obstetrics
SD	standard deviation
OR	odds ratios
CI	confidence intervals
HR	hazard ratios
SPSS	Statistical Package for the Social Sciences
