# Supplementary material for: Prognostic Value of p53 Status in Endometrial Cancer: Real-World Evidence from a Tertiary Center
Source: Cancers (Basel). 2026 Jun 1;18(11):1805. doi: 10.3390/cancers18111805 (PMC13257290; doi:10.3390/cancers18111805)
Supplement: Supplementary file 1 [file cancers-18-01805-s001.zip › cancers-4285718-supplementary.pdf]

cancers

MDPI

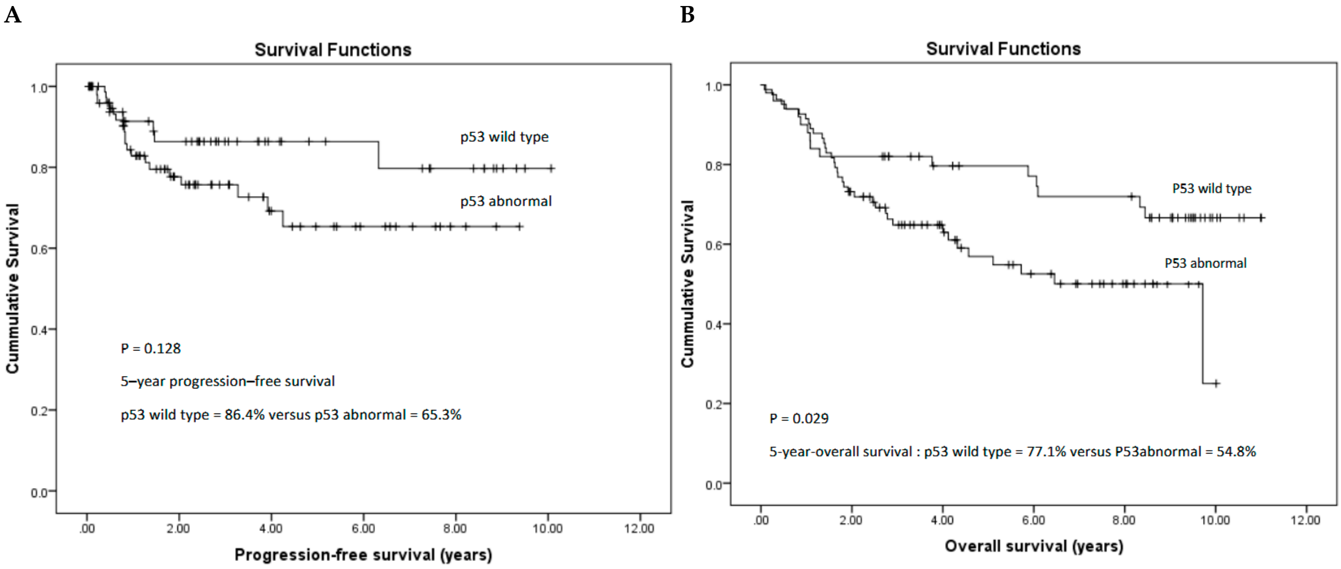

Figure S1. Kaplan–Meier curves for survival outcomes according to p53 status. (A) Kaplan–Meier analysis of progression-free survival (PFS) according to p53 status. Patients with p53 wild-type tumors showed a trend toward longer PFS compared with those with p53-abnormal tumors; however, the difference did not reach statistical significance (log-rank test, $p = 0.128$). The estimated 5-year PFS rates were 86.4% for the p53 wild-type group and 65.3% for the p53-abnormal group. (B) Kaplan–Meier analysis of overall survival (OS) according to p53 status. Patients with p53 wild-type tumors demonstrated significantly improved survival compared with those with p53-abnormal tumors (log-rank test, $p = 0.029$). The estimated 5-year OS rates were 77.1% for the p53 wild-type group and 54.8% for the p53-abnormal group. Tick marks indicate censored observations.

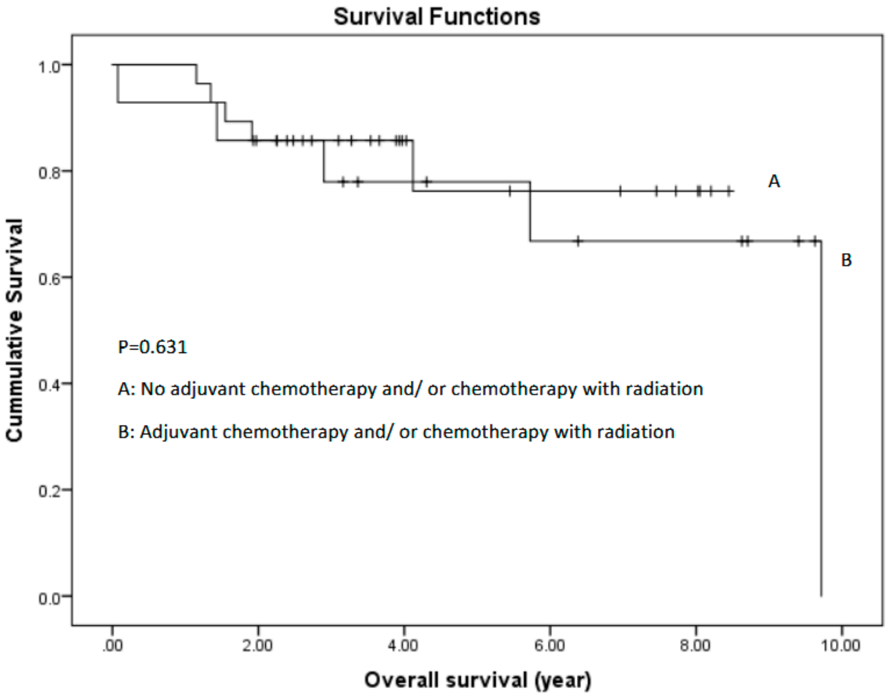

Figure S2. Overall survival according to adjuvant treatment in patients with early-stage p53-abnormal endometrial cancer. Kaplan–Meier curves showing overall survival among patients with

early-stage p53-abnormal endometrial cancer according to receipt of adjuvant chemotherapy with or without radiotherapy. Patients receiving adjuvant treatment demonstrated a numerically higher 5-year overall survival compared with those who did not receive chemotherapy (76.2% vs 66.8%); however, the difference was not statistically significant (log-rank $p = 0.631$).

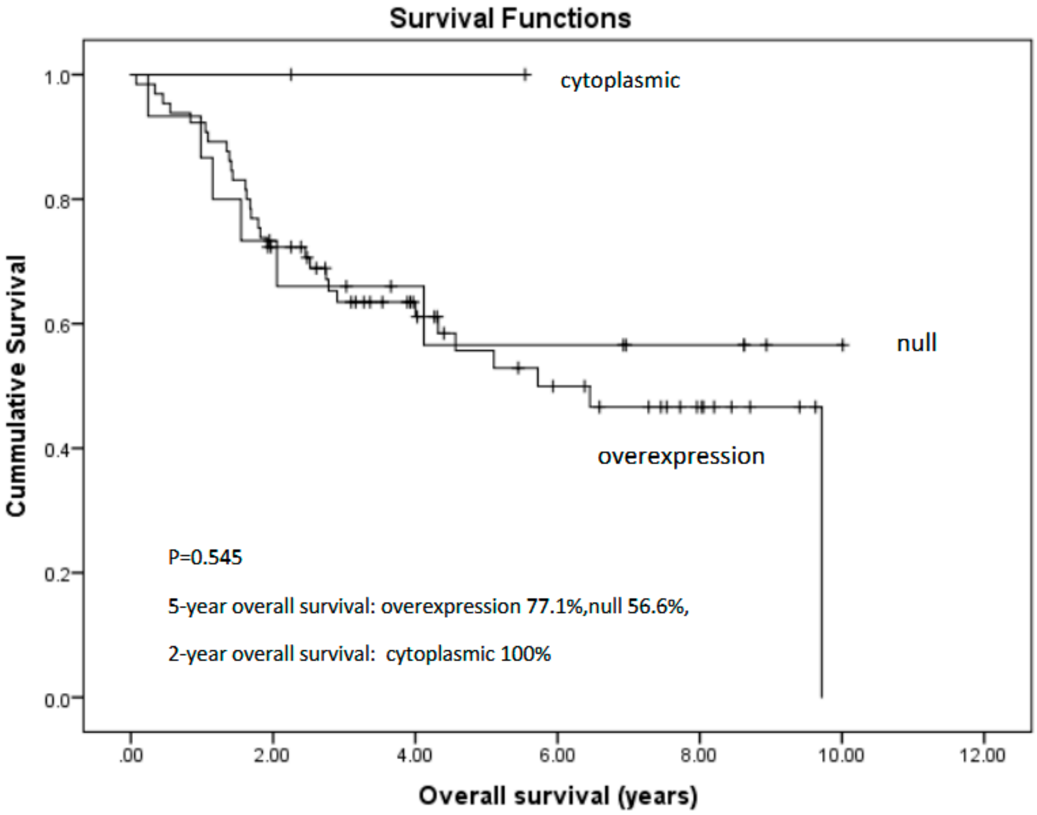

Figure S3. Kaplan–Meier curves for overall survival according to p53-abnormal staining patterns. Kaplan–Meier analysis of overall survival (OS) according to p53-abnormal immunohistochemical staining patterns, including overexpression, null, and cytoplasmic subtypes. No statistically significant differences in OS were observed among the three groups (log-rank test, $p = 0.545$). The estimated 5-year OS rates were 77.1% for the overexpression pattern and 56.6% for the null pattern, while the cytoplasmic subgroup demonstrated a 2-year OS of 100%. Tick marks indicate censored observations.

Table S1. Clinicopathologic correlates of p53-abnormal status: univariable and multivariable logistic regression analyses.

Factors	P53 Wild	P53 Abnormal	Total	Unadjusted OR (95%CI)*	P value	Adjusted OR (95%CI)**	P value
**Age**							
<=60	33(62.3)	20(37.7)	53(40.2)	**6.018 (2.780-13.026)**	**<0.001**	**7.833 (3.169-19.358)**	**<0.001**
>60	17(21.5)	62(78.5)	79(59.8)				
**BMI**							
<25	32(44.4)	40(55.6)	72(54.5)	**1.867 (0.907-3.842)**	**0.106**	**3.220 (1.303-7.955)**	**0.011**
>=25	18(30.0)	42(70.0)	60(45.5)				
**History of other malignancies**							
None	46(41.4)	65(58.6)	111(84.1)	**5.373 (1.174-24.598)**	**0.017**	**4.078 (1.099-15.142)**	**0.036**
Present	4(19.0)	17(81.0)	21(15.9)				
**Residual disease**							

None	45(37.8)	74(62.2)	119(90.2)	**0.973 (0.300-3.157)**	1.000	-	-
Present	5(38.5)	8(61.5)	13(9.8)				
**Myometrial invasion**							
Less than 50%	24(41.4)	34(58.6)	58(43.9)	**1.303 (0.642-2.645)**	0.476	-	-
More than or equal to 50%	26(35.1)	48(64.9)	74(56.1)				
**LVSI**							
None	25(32.9)	51(67.1)	76(57.6)	**0.608 (0.298-1.238)**	0.205	**0.517 (0.208-1.284)**	0.155
Present	25(50.0)	31(55.4)	56(42.4)				
**Peritoneal washing**							
Negative	48(41.0)	69(59.0)	117(88.6)	**4.522 (0.976-20.958)**	0.048	**2.688 (0.454-15.907)**	0.276
Positive	2(13.3)	13(86.7)	15(11.4)				
**Figo Stage (2023)**							
I&II	33(44.0)	42(56.0)	75(56.8)	**1.849 (0.893-3.828)**	0.106	**2.780 (1.024-7.544)**	0.045
III&IV	17(29.8)	40(70.2)	57(43.2)				

* Chi-square or Fisher's exact test **Binary regression (enter method). Variables with $p < 0.25$ in univariate analysis were included in the multivariable logistic regression model using the enter method.

Table S2. Distribution of molecular testing and results in endometrial cancer (N = 132).

Molecular testing performed	N(%)	Result distribution n (%)
p53 only	54(40.9)	Abnormal p53: 54 (100)
MMR + p53	70(53.0)	dMMR + p53 wild type: 45 (64.3)<br>pMMR + p53 abnormal: 25 (35.7)
p53 + POLE	3(2.3)	p53 abnormal + POLE negative: 2 (66.7)<br>p53 wild type + POLE positive: 1 (33.3)
MMR+p53+POLE	5(3.8)	dMMR + p53 wild type + POLE positive: 1 (20.0)<br>p53 wild type + POLE positive: 1 (20.0)<br>pMMR + p53 wild type + POLE negative: 3 (60.0)

MMR and POLE testing were not performed in all patients. Percentages were calculated within each testing group.

Table S3. Sensitivity analysis: univariable and multivariable Cox proportional hazards analyses of prognostic factors for overall survival (N = 132).

	Total (%)	5-year OS(%)	Unadjusted HR (95%CI)	P value	Adjusted HR* (95%CI)	P value
**Age**						
<=60	53(40.2)	71.1	1.510 (0.841-2.709)	0.167	1.422 (0.720-2.807)	0.310
>60	79(59.8)	60.0				
**Body mass index (kg/m^2)**						
<25	72(54.5)	65.2	0.908 (0.523-1.578)	0.733	-	-
>=25	60(45.5)	65.2				
**History of other malignancy**						
None	111(84.1)	**65.4**	1.045 (0.468-2.333)	0.914	-	-
present	21(15.9)	**66.7**				
**Underlying disease**						
None	37(28.0)	**85.5**	**2.272 (1.098-4.703)**	**0.027**	**2.143 (0.936-4.907)**	**0.071**
Present	95(72.0)	**58.9**				

**Residual disease**						
None	119(90.2)	**70.2**	**4.602 (2.265-9.347)**	**<0.001**	**2.554 (1.171-5.573)**	**0.018**
Present	13(9.8)	**30.8**				
**Myometrial invasion**						
Less than 50%	58(43.9)	**85.9**	**3.227 (1.685-6.180)**	**<0.001**	**2.332 (1.062-5.120)**	**0.035**
>=50%	74(56.1)	**51.2**				
**Lymphovascular space invasion**						
None	76(57.6)	**71.9**	**1.310 (0.756-2.269)**	0.335	-	-
Yes	56(42.2)	**59.0**				
**Tumor grade**						
Grade 1&2	30(22.70	**84.8**	**3.119 (1.322-7.357)**	**0.009**	2.198 (0.717-6.737)	0.168
Grade 3	102(77.3)	**56.1**				
**Peritoneal washing**						
Negative	117(88.6)	**70.6**	**4.018 (2.028-7.959)**	**<0.001**	2.119 (0.947-4.743)	0.068
Positive	15(11.4)	**31.1**				
**p53**						
Wild	50(37.9)	**79.7**	**1.966 (1.060-3.647)**	**0.032**	0.717 (0.293-1.752)	0.466
Abnormal	82(62.1)	**56.9**				
**FIGO Stage (2023)**						
Early (I&II)	75(56.8)	**85.7**	**4.301 (2.348-7.877)**	**<0.001**	**2.364 (1.084-5.155)**	**0.031**
Advanced (III&IV)	57(43.2)	**42.2**				
**Neoadjuvant chemotherapy**						
No	119(90.2)	**70.5**	**2.920 (1.410-6.045)**	**0.004**	2.216 (0.958-5.125)	0.063
Yes	13(9.8)	**25.6**				
**Adjuvant chemotherapy alone**						
No	97(73.5)	**70.2**	**1.983 (1.097-3.583)**	**0.023**	**0.269 (0.103-0.708)**	**0.008**
Yes	35(26.5)	**56.4**				
**Adjuvant radiation ± chemotherapy**						
No	73(55.3)	**71.2**	**1.768 (1.016-3.076)**	**0.044**	**4.384 (1.909-10.018)**	**<0.001**
yes	59(44.7)	**60.6**				

OS, overall survival; HR, hazard ratio; 95%CI, 95% confidence interval. * Variables with $p < 0.25$ in univariable analysis were included in the multivariable Cox regression model using the enter method.
